# Stratification, nitrogen fixation, and cyanobacterial bloom stage regulate the planktonic food web structure

**DOI:** 10.1111/gcb.14546

**Published:** 2019-01-09

**Authors:** Natalie Loick‐Wilde, Igor Fernández‐Urruzola, Elvita Eglite, Iris Liskow, Monika Nausch, Detlef Schulz‐Bull, Dirk Wodarg, Norbert Wasmund, Volker Mohrholz

**Affiliations:** ^1^ Leibniz Institute for Baltic Sea Research Warnemuende Rostock Germany; ^2^ Present address: Millennium Institute of Oceanography (IMO) University of Concepcion Concepción Chile

**Keywords:** amino acids, Baltic Sea, bloom stage, cyanobacteria, food web structure, mesozooplankton, N_2_ fixation, stable nitrogen isotopes, stratification

## Abstract

Changes in the complexity of planktonic food webs may be expected in future aquatic systems due to increases in sea surface temperature and an enhanced stratification of the water column. Under these conditions, the growth of unpalatable, filamentous, N_2_‐fixing cyanobacterial blooms, and their effect on planktonic food webs will become increasingly important. The planktonic food web structure in aquatic ecosystems at times of filamentous cyanobacterial blooms is currently unresolved, with discordant lines of evidence suggesting that herbivores dominate the mesozooplankton or that mesozooplankton organisms are mainly carnivorous. Here, we use a set of proxies derived from amino acid nitrogen stable isotopes from two mesozooplankton size fractions to identify changes in the nitrogen source and the planktonic food web structure across different microplankton communities. A transition from herbivory to carnivory in mesozooplankton between more eutrophic, near‐coastal sites and more oligotrophic, offshore sites was accompanied by an increasing diversity of microplankton communities with aging filamentous cyanobacterial blooms. Our analyses of 124 biotic and abiotic variables using multivariate statistics confirmed salinity as a major driver for the biomass distribution of non‐N_2_‐fixing microplankton species such as dinoflagellates. However, we provide strong evidence that stratification, N_2_ fixation, and the stage of the cyanobacterial blooms regulated much of the microplankton diversity and the mean trophic position and size of the metabolic nitrogen pool in mesozooplankton. Our empirical, macroscale data set consistently unifies contrasting results of the dominant feeding mode in mesozooplankton during blooms of unpalatable, filamentous, N_2_‐fixing cyanobacteria by identifying the at times important role of heterotrophic microbial food webs. Thus, carnivory, rather than herbivory, dominates in mesozooplankton during aging and decaying cyanobacterial blooms with hitherto uncharacterized consequences for the biogeochemical functions of mesozooplankton.

## Graphical abstract

R/V Meteor passing a decaying bloom of unpalatable, filamentous, N_2_‐fixing cyanobacteria in the Baltic Sea in August 2015 that are expected to increase in large areas of the global ocean in the future. While herbivory dominated the trophic structure of mesozooplankton in earlier bloom stages, direct estimates of carnivory in field mesozooplankton identified the at times important role of heterotrophic microbial food webs namely in such aging and decaying cyanobacterial blooms with hitherto uncharacterized consequences for the biogeochemical functions of mesozooplankton. Photo: Andreas Raeke, DWD.

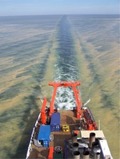

## 1 INTRODUCTION

Approximately 93% of the excess heat energy trapped since the 1970s has been absorbed into the oceans, leading to a variety of changes in ocean conditions (Jewett & Romanou, 2017 and references therein). The most rapid warming of the mean sea surface temperature (>0.9°C) is observed in land‐locked or semienclosed seas such as the Baltic Sea, North Sea, Black Sea, Japan Sea/East Sea, and East China Sea and over the Newfoundland–Labrador Shelf (Belkin, 2009). The large heat absorption alters the fundamental physical properties of the ocean by increasing the stratification of the water column (Jewett & Romanou, 2017 and references therein). This process indirectly impacts chemical and biological properties such as changing nitrogen and carbon biogeochemical cycles that can result in increasing standing stocks of filamentous, N_2_‐fixing cyanobacteria such as *Trichodesmium* (Gattuso et al., 2015; Hutchins & Fu, 2017; Jewett & Romanou, 2017; Paerl & Huisman, 2008; Roy et al., 2011). So far, we lack a consistent understanding of how stratification and blooms of unpalatable, N_2_‐fixing, filamentous cyanobacteria affect the food web structure in aquatic ecosystems (Steinberg & Landry, 2017).

The number of trophic levels between autotrophs and mesozooplankton is a critical determinant to estimate the transfer of net primary production into the biological carbon pump and into higher trophic levels, such as fish, according to empirical data and ecosystem models (Jewett & Romanou, 2017; Peck et al., 2018; Steinberg & Landry, 2017). Direct grazing on filamentous, N_2_‐fixing cyanobacteria by mesozooplankton organisms only sporadically takes place by some specialists (Engstroem et al., 2000; Loick‐Wilde et al., 2012; O'Neil, 1999). The transfer of nutrients from unpalatable, filamentous, N_2_‐fixing cyanobacterial genera such as *Trichodesmium*,* Nodularia,* or *Aphanizomenon* into mesozooplankton is thought to be mainly indirect through grazing on a microbial food web (summarized by Motwani, Duberg, Svedén, & Gorokhova, 2018; Wannicke, Korth, Liskow, & Voss, 2013). In these microbial food webs, auto‐, mixo‐, and heterotrophic microorganisms incorporate and grow on ammonium, amino acids (AA), or other dissolved organic nitrogen (DON) forms (Stal, Staal, & Villbrandt, 1999) that can be exuded in large quantities from filamentous cyanobacteria during N_2_ fixation (Adam et al., 2016; Mulholland, Bernhardt, Heil, Bronk, & O'Neil, 2006; Ploug, 2008; Stal et al., 2003). The majority of studies suggest that mesozooplankton are herbivorous when filamentous, N_2_‐fixing cyanobacteria dominate a microplankton community because the animals rely on co‐occurring autotrophic phytoplankton species (Hannides, Popp, Landry, & Graham, 2009; McClelland, Holl, & Montoya, 2003; Mompeán, Bode, Gier, & McCarthy, 2016). All of these trophic studies used nitrogen stable isotope ratios in AAs to identify the mean trophic position (TP) of mixed field mesozooplankton samples without experimental manipulations (Chikaraishi et al., 2009; McClelland & Montoya, 2002; Steffan et al., 2015). Only recently, a study showed that mesozooplankton at times can also be carnivorous during a filamentous, N_2_‐fixing cyanobacterial bloom according to TP estimates (Eglite et al., 2018). Different from other studies this bloom was in a very late, decayed stage but with surprisingly fast amino acid turnover during N_2_ fixation by the remaining intact cells (Loick‐Wilde et al., 2018).

The TP approach is based on the stable nitrogen isotope composition (δ^15^N) of the AAs phenylalanine (Phe) and glutamic acid (Glu) but sometimes also on phenylalanine (Phe) and alanine (Ala, reviewed by Ohkouchi et al., 2017). The δ^15^N of Phe is determined during its synthesis by autotrophs from an inorganic nitrogen source, and its isotopy remains virtually unaltered across trophic levels because no carbon–nitrogen atomic bonds are cleaved during the metabolism of essential Phe in heterotrophs (Chikaraishi et al., 2009; McClelland & Montoya, 2002; Steffan et al., 2015). The δ^15^N‐Phe in heterotrophs such as mesozooplankton is therefore a proxy for the dominant inorganic nitrogen source (e.g., nitrate or N_2_) in an aquatic food web (Eglite et al., 2018; McClelland & Montoya, 2002; McMahon, McCarthy, Sherwood, Larsen, & Guilderson, 2015; Sherwood, Lehmann, Schubert, Scott, & McCarthy, 2011). Glu and Ala belong to a group of AAs called “trophic AAs.” Their isotopic signatures undergo large isotopic fractionation during the incorporation of a diet by a consumer (Montoya, Carpenter, & Capone, 2002; Steffan et al., 2015). In combination, Phe and Glu are the pair most often used for the stepless determination of the mean TP (TP_Glu/Phe_) of a mixed field plankton sample without experimental manipulations (Chikaraishi et al., 2009; McClelland & Montoya, 2002; Ohkouchi et al., 2017), while the mean TP based on the δ^15^N‐Ala and δ^15^N‐Phe (TP_Ala/Phe_) was recently suggested to better resolve the protist level (Décima, Landry, Bradley, & Fogel, 2017).

The Baltic Sea is one of the largest brackish ecosystems in the world and overall not a nutrient‐poor sea; rather, it experiences over fertilization (Conley, 2012; Voss et al., 2011). However, in midsummer every year, its dissolved nitrate and phosphorus pools are largely depleted, and during this time, massive blooms of unpalatable, N_2_‐fixing, filamentous cyanobacteria, namely, *Nodularia* and *Aphanizomenon,* occur (Kahru, Elmgren, Di Lorenzo, & Savchuk, 2018; Karlson et al., 2015; Wasmund, 1997). In addition, the Baltic Sea faces warming already today (Belkin, 2009; Kahru, Elmgren, & Savchuk, 2016; Suikkanen et al., 2013). This scenario leads to an increased summer surface temperature and stratification, which are among the primary variables that regulate variations in the intensity and occurrence of *Nodularia* and *Aphanizomenon* blooms in the offshore waters of the central Baltic Sea (Kahru & Elmgren, 2014; Kononen et al., 1996; Vahtera et al., 2007; Wasmund, 1997). Despite large structural (e.g., average water depths of only 58 m) and functional (e.g., low rate of exchange with North Atlantic waters and predominantly brackish conditions) differences between the Baltic Sea compared to marine coastal and offshore ecosystems, these regular and extensive cyanobacterial blooms offer the opportunity to develop a more mechanistic understanding of the effect of stratification and unpalatable, N_2_‐fixing, filamentous cyanobacterial blooms on the number of trophic levels between autotrophs and mesozooplankton (Reusch et al., 2018).

In this study, we empirically identified how the trophic structure in planktonic food webs changed along with different abiotic and biotic factors across the Baltic Sea in summer. We determined the impact of N_2_ fixation and the trophic structure of planktonic food webs according to two AA nitrogen stable isotope‐based biogeochemical proxies (δ^15^N‐Phe and TP_Glu/Phe_) from two mesozooplankton size fraction samples from stations across the Baltic Sea. The proxies were connected to the results from Eglite et al. (2018) and Loick‐Wilde et al. (2018) and to a large set of environmental variables, for example, mixed layer depth, nutrient concentrations, N_2_ fixation rates (in parts from Loick‐Wilde et al., 2018), microplankton cell carbon concentrations and biodiversity, and the sizes of the bulk and metabolic nitrogen pools in mesozooplankton. This process allowed us to conceptualize how the mean TP_Glu/Phe_ of mesozooplankton can change along with the stratification, N_2_ fixation, and filamentous cyanobacterial bloom stage in an aquatic ecosystem.

## 2 MATERIALS AND METHODS

**Figure 1:**
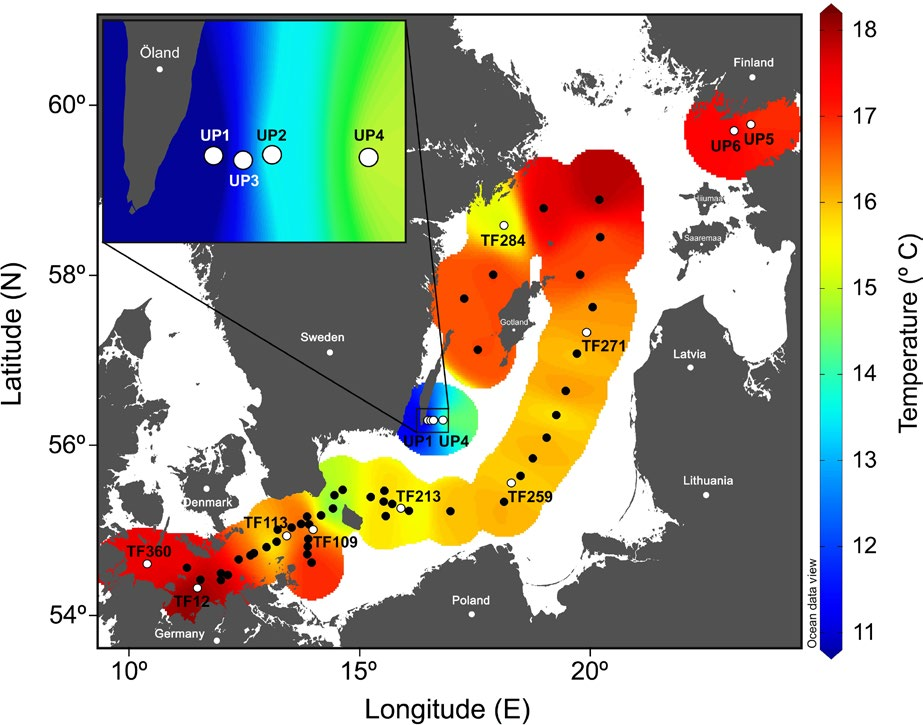
Station map superimposed on the sea surface temperature across the Baltic Sea during RV Meteor cruise M117 in July/August 2015. Biogeochemical variables were taken at 14 stations (marked in white), of which 11 contained all 124 variables for a detailed multivariate analysis including three upwelling stations off Öland (UP2‐UP4), two upwelling stations in the Gulf of Finland (UP5 and UP6), one western Baltic Sea station (TF12), and five central Baltic Sea stations (TF113, TF109, TF213, TF259, and TF271).

Samples were collected on a transect including a total of 59 hydrographical stations (Stas.) across the Baltic Sea in July and August 2015 during cruise M117 on board the RV *Meteor*. Fourteen stations were sampled for biogeochemical variables (Figure 1). Hydrographic data and water samples for chlorophyll a (Chl. a) and nutrient determinations were obtained by deployment of a Seabird SBE‐911 plus CTD equipped with oxygen and fluorescence sensors and mounted on a rosette sampler with thirteen 5‐L GO‐FLO bottles (Hydro‐Bios, Kiel, Germany). Nutrient samples were analyzed directly on board according to Rhode and Nehring (1979) and Hansen and Koroleff (2007). Additional samples of particulate organic matter (POM) were taken from the Chl. a maximum by filtering 0.5–1.0 L of seawater through precombusted Whatman GF/F filters (0.7 μm pore size, 25 mm in diameter) for elemental (total carbon and total nitrogen) analyses. All filters were stored at −20°C after shock‐freezing in liquid nitrogen (−196°C). Additionally, 200–500 mL samples of surface water (from depths of 1 m, 5 m, 10 m, 15 m, and 20 m) were filtered for Chl. a concentrations on glass‐fiber filters (Whatman GF/F) that were shock‐frozen in liquid N_2_ and stored at −80°C. The 96% ethanol extracts were used for fluorometric analysis according to the guidelines of HELCOM (2017). Nano‐ and microplankton samples (summarized as microplankton) were collected from an integrated depth between 0 and 10 m at 12 stations across the Baltic Sea, including two upwelling sites (Table 1). At Stas. TF12, TF360, TF109, TF113, TF213, TF259, and TF271, additional microplankton samples from a 20 m depth were taken. All microplankton samples were fixed with acid Lugol's solution, and at least 500 counting units were counted in sedimentation chambers under the inverted microscope as described in the manual of HELCOM (2017). Microplankton organisms were assigned to genera (species, if possible) and size classes, for which the specific volume was estimated as shown by Olenina et al. (2006). From these biovolume estimates, the carbon content of the taxa (in the following cell carbon biomass) was calculated after Menden‐Deuer and Lessard (2000), as recommended by HELCOM (2017). Microplankton included autotrophic, mixotrophic, and heterotrophic species.

**Table 1:** Hydrographic characteristics, nutrient and Chl. a concentrations, and microplankton biodiversity indices (H’: Shannon‐Wiener's diversity; J_0_: Pielou's evenness; and d: Margalef's species richness) found in the surface mixed layer (ML) in July/August 2015. Dashes stand for no data. Chl. a from fluorometrical data from discrete measurements are given in mg m^−3^ and from the water column sensor in relative units, and both variables were significantly correlated (Supporting Figure S1)

Station	Sea area	*ML depth	*Temperature	*Salinity	*Oxygen	*PO_4_^3−^	*SiO_2_	*DIN	NH_4_^+^	Chl. a	*Chl. a	*H’	*J_o_	*d
		(m)	(ºC)	(PSU)	(mL L^−1^)	(μM)				(mg m^−3^)	(rel. units)			
TF360	wB	13.3	17.6	15.1	3.8	–	–	–	–	–	1.9	3.6	0.67	9.0
TF12*	wB	11.0	17.9	10.8	6.2	0.13	10.5	0.11	0.36	–	1.4	3.8	0.67	10.4
TF113*	AB	16.5	16.3	8.3	6.5	0.19	11.2	0.06	0.35	2.1	1.6	3.6	0.69	7.9
TF109*	AB	17.0	16.1	7.9	6.6	0.27	11.5	0.07	0.28	–	1.6	3.5	0.68	8.1
TF213*	BB	27.8	15.6	7.6	6.4	0.28	13.2	0.07	0.29	2.3	1.4	4.0	0.76	9.1
TF259*	sGB	27.8	15.9	7.2	6.5	0.33	17.4	0.05	0.26	2.9	1.4	3.9	0.76	8.7
TF271*	eGB	22.3	16.3	7.8	6.5	0.04	9.9	0.10	0.34	3.2	2.4	3.8	0.75	7.6
TF284	wGB	9.8	15.2	5.7	7.1	0.02	8.8	0.12	0.29	3.6	1.9	–	–	–
UP1	ÖL	8.0	8.7	7.0	7.2	0.56	14.2	0.15	–	1.1	0.6	–	–	–
UP2*	ÖL	8.3	14.1	6.9	6.7	0.28	12.3	0.12	–	1.5	1.0	3.2	0.72	7.0
UP3*	ÖL	5.8	11.6	6.9	6.8	0.38	12.6	0.19	–	1.2	0.9	2.6	0.61	6.2
UP4*	ÖL	10.4	15.9	6.8	6.6	0.13	12.1	0.14	–	1.8	1.0	3.3	0.73	6.7
UP5*	GoF	8.5	16.9	6.0	6.5	0.16	10.3	0.19	–	3.2	1.8	2.7	0.56	6.0
UP6*	GoF	10.3	17.5	6.1	6.5	0.06	9.2	0.24	–	4.4	2.5	3.0	0.63	5.5

^*^Stations and variables (among others) included in the principal component analysis. Abbreviations: wB: western Baltic; AB: Arkona Basin; BB: Bornholm Basin; sGB: southern Gotland Basin, eGB: eastern Gotland Basin; wGB: western Gotland Basin; ÖL: off Öland; GoF: Gulf of Finland. DIN: dissolved inorganic nitrogen from nitrate and nitrite.

N_2_ fixation rates into particulate organic nitrogen were measured according to Montoya, Voss, Kaehler, and Capone (1996) at the upwelling Stas. UP2, UP3, and UP4 and were combined with published rates from Stas. TF109, TF213, TF259, and TF271 in the central Baltic Sea (Loick‐Wilde et al., 2018). Details of the experiments can be found in Loick‐Wilde et al. (2018).

Zooplankton were collected by vertical tows through surface waters using a UNESCO WP‐2 net (0.25 m^2^ mouth opening) fitted with a 100 μm mesh size at 14 stations (Table 2). Once on deck, the mesozooplankton were concentrated in a light trap for 3 h before further processing to avoid sample contamination with phytoplankton cells. Then, the samples were divided into 100–300 μm and >300 μm size groups by sieving the samples through a 300 μm nylon mesh and stored in the manner described for the aforementioned filters. Plankton samples were then analyzed in the laboratory for total organic nitrogen (TN) and carbon (TC), and AA composition, as well as for bulk and compound‐specific isotope ratios.

**Table 2:** δ^15^N values (‰) of bulk nitrogen and of individual amino acids and the heterotrophic resynthesis index (ΣV) in the two mesozooplankton size fractions collected from the upper water column across the Baltic Sea in July/August 2015

Station	Depth (m)	Bulk	Trophic AA							Source AA			Metabolic AA	∑V
			Ala	Asp	Glu	Ile	Leu	Pro	Val	Gly	Lys	Phe	Thr	
		(*δ* ^15^N relative to atmospheric N_2_)												
***Zoo***_***100–300 μm***_														
TF360	0–15	5.6	12.8	10.8	15.8	10.5	10.8	10.7	13.2	3.6	7.0	5.0	−2.3	1.6
TF12	0–25	6.1	11.2	10.8	15.7	12.9	8.9	10.3	10.3	3.0	6.0	5.0	−0.9	1.6
TF113	0–20	4.6	10.7	9.7	12.3	6.6	7.7	9.1	10.0	−07	3.1	1.7	−4.7	1.4
TF109	0–43	3.5	10.7	7.8	12.7	7.7	8.5	8.8	11.3	−0.3	1.9	−0.8	−1.7	1.5
TF213	0–25	2.1	9.8	7.1	9.9	9.2	6.9	5.9	10.0	−1.8	1.2	−2.3	−6.8	1.5
TF259	0–20	4.8	14.4	11.5	14.8	12.9	10.4	9.9	13.5	−0.9	2.4	−2.6	−10.2	1.6
TF271^a^	0–25	3.7 (±0.8)	12.2(±1.9)	9.1 (±1.7)	12.9(±2.2)	9.1 (±3.3)	7.2 (±1.3)	9.9 (±1.5)	10.2(±2.2)	−2.5(±0.3)	0.9 (±0.4)	−2.1(±0.7)	−9.3 (±0.8)	1.8 (±0.1)
TF284	0–20	4.3	11.1	9.4	12.3	11.2	8.6	9.8	10.7	−0.6	2.2	−0.5	−8.1	1.0
UP1	0–15	4.6	12.6	11.1	13.4	9.4	7.9	9.4	11.1	1.1	1.9	0.0	4.4	1.5
UP2	0–53	4.5	14.1	13.0	14.0	8.5	8.5	10.3	11.0	−1.2	1.7	0.9	−6.7	2.0
UP3	0–35	4.6	12.1	9.8	13.1	8.4	7.4	9.1	9.6	−0.4	1.0	0.0	3.8	1.5
UP4	0–20	3.5	10.6	8.0	11.4	9.4	7.0	8.7	10.2	−2.3	0.0	−0.8	−6.2	1.2
UP5	0–30	6.4	12.0	11.7	13.2	12.1	9.7	12.0	13.0	−1.9	4.2	2.8	0.0	0.7
UP6	0–45	5.6	12.0	11.6	12.9	10.9	10.5	12.3	12.2	−1.0	3.7	2.7	−1.8	1.1
***Zoo***_***300 μm***_														
TF360	0–15	7.3	14.6	11.9	15.1	11.8	12.2	12.1	14.1	2.0	4.6	5.8	−0.8	1.3
TF12	0–25	7.7	17.8	15.8	18.7	15.9	14.8	14.8	17.4	3.1	5.9	5.3	−5.0	1.3
TF113	0–20	5.8	16.8	13.5	17.0	14.5	12.0	12.5	15.3	1.0	4.1	2.5	−5.9	1.6
TF109	0–43	6.7	17.2	14.6	18.0	15.9	13.3	12.6	16.0	1.3	4.1	0.5	−8.8	1.6
TF213	0–25	4.7	16.4	12.7	15.9	16.0	12.5	10.9	15.8	−0.0	3.2	−0.5	−9.0	1.9
TF259	0–28	4.7	15.8	11.7	15.7	15.5	11.6	11.3	15.4	−.5	2.0	−1.5	−10.6	2.0
TF271[Link]	0–25	5.4 (±0.1)	15.5(±0.8)	12.0(±0.8)	16.9(±0.8)	10.6(±0.8)	13.0(±0.8)	13.1(±0.8)	13.0(±0.8)	−1.3(±0.8)	1.5 (±0.8)	−1.4(±0.8)	−11.3 (±0.8)	1.9 (±0.1)
TF284	0–20	5.9	15.7	12.9	15.8	15.6	12.5	12.9	15.6	0.8	3.4	−0.5	−7.7	1.4
UP1	0–15	5.7	17.2	11.1	13.8	12.5	11.6	14.7	15.5	0.5	1.8	2.9	−7.3	1.7
UP2	0–53	5.3	15.0	13.0	15.6	13.1	10.7	13.1	13.1	0.6	3.2	2.4	−6.0	1.2
UP3	0–35	5.4	15.2	9.8	14.6	13.4	10.7	13.6	12.8	−1.3	2.7	4.7	−7.3	1.0
UP4	0–20	5.1	14.3	8.0	14.6	13.8	10.4	12.0	13.6	−1.3	2.5	−0.6	−10.6	1.6
UP5	0–30	8.3	14.9	11.7	15.3	15.0	11.7	14.1	13.6	2.1	4.6	2.6	−2.5	1.3
UP6	0–45	8.1	15.8	11.6	16.8	15.3	12.4	14.3	14.7	1.6	5.3	3.2	−2.6	1.1

a Data taken from Eglite et al. (2018) and presented as the mean values with standard deviations from 3 to 4 samples.

### 2.1 Elemental and biochemical analyses of plankton samples

TN and TC and individual concentrations of 11 AAs as well as the respective stable nitrogen isotope values in POM and mesozooplankton samples were measured in samples from the upwelling stations UP1, UP2, UP3, and UP4; the western Baltic Sea Stas. TF360 and TF12; and the central Baltic Sea Stas. TF113, TF109, TF213, TF259, and TF284 and were combined with the published data from Station (Sta.) TF271 (Eglite et al., 2018). Individual concentrations and nitrogen stable isotopes of 11 AAs included the so‐called “source” AAs glycine ‐Gly‐, lysine ‐Lys‐, and phenylalanine ‐Phe‐; the “trophic” AAs alanine ‐Ala‐, aspartic acid ‐Asp‐, glutamic acid ‐Glu‐, isoleucine ‐Ile‐, leucine ‐Leu‐, proline ‐Pro‐, and valine ‐Val‐; and the “metabolic” AA threonine ‐Thr‐, categorized by Germain, Koch, Harvey, and McCarthy (2013), McClelland and Montoya (2002), and Chikaraishi et al. (2009) according to the sensitivity of each AA to trophic enrichment in ^15^N. It should be noted that Asp and Glu also include the amide forms asparagine and glutamine, respectively, with the N isotopic signature coming only from the α‐amino‐N from both compounds as the amide N is lost during hydrolysis. Details of the elemental analysis–isotope ratio mass spectrometry (EA‐IRMS), gas chromatography–mass spectrometry (GC‐MS), and gas chromatography–combustion–isotope ratio mass spectrometry (GC‐C‐IRMS) analyses can be found in Eglite et al. (2018) and in the Supporting Material S1.

### 2.2 Proxies derived from amino acid analyses

The δ^15^N values of Phe from the two mesozooplankton size fractions were used as time‐integrating proxies for the dominant inorganic nitrogen source sustaining the different planktonic food webs across the Baltic Sea (Eglite et al., 2018; McClelland & Montoya, 2002; McMahon et al., 2015; Sherwood et al., 2011). Different from the δ^15^N‐Phe in POM, the δ^15^N‐Phe in zooplankton integrates over transient events that can alter the isotopic baseline in autotrophs as described for bulk δ^15^N values in POM and thus provides a more robust proxy for the inorganic N source (summarized by Loick‐Wilde et al., 2016).

The proportion of Phe from N_2_ fixation in mesozooplankton can be estimated according to a modified two‐source mixing model after Montoya et al. (2002). For this model, it is assumed that the δ^15^N‐Phe value of the mesozooplankton is a mixture of the δ^15^N values of Phe that is synthesized by phytoplankton from the two new nitrogen sources nitrate and nitrogen from N_2_ fixation (diazotroph N). For this estimation, the δ^15^N‐Phe value based on its synthesis from nitrate and the δ^15^N‐Phe value based on its synthesis from N_2_ fixation must be known. Here, we assume that the Phe in Baltic Sea zooplankton based on a synthesis from nitrate (δ^15^N‐Phe_Nitrate_) is at least 2.6‰ as found in zooplankton in the subtropical North Pacific (McCarthy, Benner, Lee, & Fogel, 2007). The justification for this is that subsurface nitrate has the same δ^15^N value of 3.5 ‰ in both ecosystems (Casciotti, Trull, Glover, & Davies, 2008; Korth, Deutsch, Frey, Moros, & Voss, 2014). The δ^15^N‐Phe value in Baltic Sea zooplankton based on a synthesis from N_2_ fixation (δ^15^N‐Phe_Diazo_) is assumed to be −3.6‰ as found for *Trichodesmium* (McClelland et al., 2003). The contribution of diazotroph Phe to mesozooplankton Phe (% Diazo Phe‐N) was calculated as follows:

(1) $$\%\;\text{Diazo Phe - N}=100\times {\left(\frac{\delta^{15}{\text{N - Phe}}_{\text{Zoo}}-\delta^{15}{\text{N - Phe}}_{\text{Nitrate}}}{\delta^{15}{\text{N - Phe}}_{\text{Diazo}}-\delta^{15}N{\;\text{- Phe}}_{\text{Nitrate}}}\right)}^{}$$

where δ^15^N‐Phe_Zoo_ is the δ^15^N‐Phe value of one of the two mesozooplankton size fractions, and the other variables are the two δ^15^N‐Phe endmember values as defined above.

The δ^15^N values of Glu and Phe together were used to estimate the mean trophic position of mesozooplankton samples based on equation (2) according to Chikaraishi et al. (2009):

(2) $$\text{TP}=\frac{\delta^{15}\text{N - Glu}-\delta^{15}\text{N - Phe}+\left|\beta \right|}{\text{TDF}}+\lambda$$

where δ^15^N‐Glu and δ^15^N‐Phe are the measured values from a single sample, and *β* is the δ^15^N difference between Glu and Phe in the primary producers (−3.4 ± 0.9‰ for aquatic cyanobacteria and algae). TDF represents the trophic discrimination factor between Glu and Phe (7.6 ± 1.2‰) at each trophic shift between a consumer and its diet, and *λ* represents the first trophic level (=1) of the food web. Accordingly, a TP of 1.0 ± 0.3 represents the dominance of autotrophs in a mixed sample, a TP of 2.0 ± 0.3 represents the dominance of herbivores, and a TP of 3.0 ± 0.3 represents the dominance of carnivores (Chikaraishi et al., 2014; Eglite et al., 2018; McMahon & McCarthy, 2016).

TPs based on δ^15^N‐Ala and δ^15^N‐Phe (Décima et al., 2017) and a proxy for heterotrophic resynthesis (ΣV) that tracks to what degree the δ^15^N‐AA values in mesozooplankton samples were modified by heterotrophic microbial alterations (McCarthy et al., 2007; Mompeán et al., 2016) have also been calculated. However, no significant differences between either the TP estimation approach or no indication for heterotrophic resynthesis according to ΣV values of maximum 2 (Table 2) and coupled ΣV and TP values (Ohkouchi et al., 2017) were found (Supporting Figure S3). Therefore, we proceeded with the TP_Glu/Phe_ estimates (in short TP) for both mesozooplankton size fractions.

The sum of nitrogen in AAs was used as a measure of the metabolic nitrogen pool size in mesozooplankton according to Mayzaud and Conover (1988). The metabolic nitrogen pool is a critical measure to estimate the availability of AAs as precursors for other macromolecules, such as nucleotides or fatty acids, or as an energy pool in mesozooplankton (Mayzaud & Conover, 1988). Normalization of the total hydrolyzable amino acid nitrogen (THAAN) concentration to total organic nitrogen (TN) as the wt% TN‐specific THAA nitrogen concentrations (*μ*g THAAN (100 *μ*g TN)^−1^ or THAAN wt%TN) was performed for comparability of the THAAN pools in the two mesozooplankton size fractions across the different stations (Cowie & Hedges, 1992).

### 2.3 Estimation of mixed layer depth

For the mixed layer depth (MLD) estimation, the density anomaly σ_T_ was calculated according to TEOS10 (International Thermodynamic Equation of Seawater‐2010). Standard MLD estimation methods applied in oceanic conditions (e.g., Kara, Rochford, & Hurlburt, 2000; Thomson & Fine, 2003) failed for some profiles of the data set due to the special conditions of the brackish Baltic Sea. Therefore, a slightly modified difference threshold method was applied. The mean σ_T_ of the upper layer was calculated stepwise down to depth z_d_, and the difference Δσ_T_(*z*_d_) to σ_T_(*z*_d_) was derived:

(3) $$\Delta \sigma_{T}\left(z_{d}\right)=\sigma_{T}\left(z_{d}\right)-\frac{1}{z_{d}}\int_{z_{d}}^{0}\sigma_{T}\left(z\right)dz$$

Starting at the surface, the MLD was reached when Δσ_T_(*z*_d_) exceeded a threshold of 0.1 g kg^−1^ + the standard deviation of σ_T_ in the range 0 to *z*_d_ (mixed layer). The results were verified by visual inspection of the density profiles.

### 2.4 Statistical analyses

A cluster analysis was used to identify cell carbon‐specific communities of microplankton taxa based on samples from the upper 10 m from 12 stations across the Baltic Sea, including two upwelling areas. A similarity (SIMPER) analysis additionally identified which taxa shaped the respective communities. Cluster analyses including the available microplankton data from 20 m depth have also been tested but did not show significant differences to the more consistent data set from the upper 10 m (Supporting Figure S2).

The diversity of the microplankton species/taxa in the upper 10 m at the different stations was estimated using Margalef's index for species richness (d), Shannon–Wiener's diversity index (H’), and Pielou's evenness index (J_0_). Notably, logarithms to base 2 were used in the calculation of H’. All biodiversity indices used were based on the microplankton cell carbon biomass (Clarke & Warwick, 2001).

A correlation‐based principal component analysis (PCA) was used to identify the biotic and abiotic factors that impact the mean trophic position of the two mesozooplankton size fractions across the Baltic Sea during blooms of unpalatable, filamentous, N_2_‐fixing cyanobacteria (Clarke & Warwick, 2001). The PCA included a total of 124 normalized environmental variables, of which the majority belonged to the 81 individually counted microplankton taxa. Additionally, microplankton taxa were summarized into 22 groups to resolve higher taxonomic levels (mainly orders) as well as the categories unicellular cyanobacteria, filamentous cyanobacteria, or flagellates (Supplemental Data SD1_1 and SD2_2). All 124 variables were available for 11 stations across the Baltic Sea, excluding Stas. TF360 (not sampled for nutrients), UP1, and TF284 (not sampled for microplankton). The number of factors (or principal components, PCs) included in the PCA was based on the a priori criterion to cover the abiotic variables from Table 1 that were included in the PCA and that typically explain much of the biotic variability in an aquatic ecosystem (e.g., Raes et al., 2018 and references therein). This result was crosschecked and confirmed with a scree test (Bryant & Yarnold, 1995). The leading abiotic and biotic variables of a PC were chosen according to eigenvectors above the cut‐point of ǀ0.100ǀ and were mainly attributed to the leading PC for which the eigenvector above ǀ0.100ǀ was highest. Post hoc linear correlations between the leading biotic variables and the leading abiotic variables of a PC further elucidated these relationships especially for biotic variables with eigenvectors above ǀ0.100ǀ in more than one of the leading factors. Post hoc linear correlation was further used to identify the leading variables that directly correlated with the mean TPs (TP_100_ and TP_300_) and the proxies for the dominant inorganic nitrogen source (δ^15^N‐Phe_100_ and δ^15^N‐Phe_300_) of the two mesozooplankton size fractions, which were of special interest for this study.

All biodiversity indices, community similarities, and the PCA were analyzed using PRIMER‐6 Software (Primer‐E Ltd., UK).

The data sets of N_2_ fixation rates from three upwelling stations (UP2, UP3, and UP4) and Sta. TF284 together with the published rates at Stas. TF109, TF213, TF259, and TF271 from Loick‐Wilde et al. (2018) were too small to be included in the PCA. Therefore, the correlation of the N_2_ fixation rates with the different abiotic and biotic variables was analyzed separately by regression analyses. Furthermore, three missing THAAN values for Stas. UP1 and UP5 (poor recovery of internal standards in the GC‐MS runs) precluded the inclusion of the THAAN data into the PCA, and therefore, the correlation of the THAAN with the different abiotic and biotic variables was also analyzed separately.

## 3 RESULTS

### 3.1 Environmental conditions

A typical thermal stratification was found in the Baltic Sea in July/August 2015, with a subsurface thermocline that separated the warm surface waters from the colder intermediate layer. The inflow of saline water from the Kattegat area into the central Baltic Sea resulted in the characteristic west to east salinity decrease (Table 1). The sea surface temperatures ranged from 15.6°C to 17.9°C, excluding the upwelling area off Öland (Figure 1). The stations referred to here as upwelling stations exhibited clearly lower temperatures and/or shallower thermoclines, especially the ones located off Öland. In the central Baltic Sea, notable temperature and mixed layer depth gradients occurred with lower temperatures and deeper mixed layer depths in the eastern and southern Gotland and Bornholm Basins (Stas. TF271, TF259, and TF213) compared to that in the Arkona Basin (Stas. TF109 and TF113, Table 1, Figure 1).

Dissolved, oxidized, inorganic nitrogen concentrations of nitrate and nitrite (DIN) were relatively low at all stations and ranged from 0.05 to 0.24 μM with lowest concentrations at the central Baltic Sea Stas. TF109, TF113, TF213, TF259, and TF271 (average of 0.07 ± 0.02 μM, Table 1). Upwelling stations had slightly higher DIN concentrations than most other stations, with DIN values between 0.12 μM and 0.24 μM (average of 0.17 ± 0.04 μM, Table 1) that were double the concentrations measured in the central Baltic basins. Phosphate concentrations had an average value of 0.22 ± 0.15 μM, and the lowest values coincided with the highest Chl. a concentrations at the eastern and western Gotland stations (Stas. TF271 and TF284) and at the upwelling Sta. UP6 in the Gulf of Finland (Table 1). The lowest Chl. a values were found at the upwelling stations off Öland.

Volumetric N_2_ fixation rates at the upwelling stations were very low, yet detectable, at 0.05–0.1 nmol L^−1^ h^−1^ at the upwelling Stas. UP2 and UP3 and were enhanced at 0.8 nmol L^−1^ h^−1^ further offshore at Sta. UP4 (Supporting Data SD2_2). In the central Baltic Sea, the rates ranged from 2.6 nmol L^−1^ h^−1^ to 9.3 nmol L^−1^ h^−1^ in the Chl. a maxima of the mixed layer (rates after 24 h of incubation from Loick‐Wilde et al., 2018). Linear regression analyses identified positive correlations of volumetric N_2_ fixation rates with Chl. a concentrations and the cell carbon biomasses of N_2_‐fixing Nostocales (namely, *Nodularia* and *Aphanizomenon*) (Supporting Data SD2_1).

**Figure 2:**
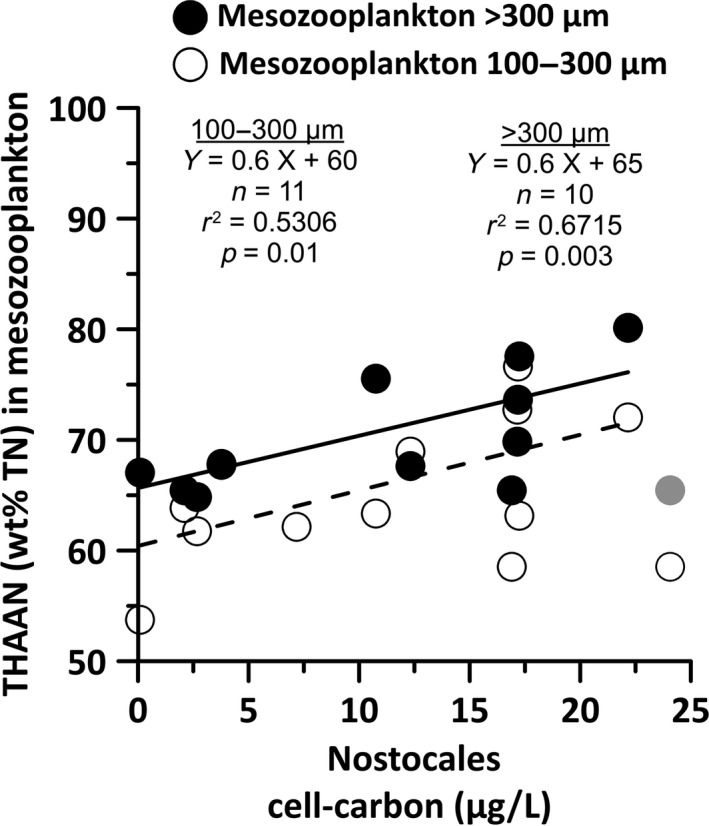
Total hydrolyzable amino acid nitrogen concentrations (THAAN in wt% TN) in two mesozooplankton size fractions relative to the cell carbon concentrations of Nostocales (μg cell‐C L^−1^) averaged from the upper 10 m (UP stations) or upper 20 m (other stations). The regression statistics for the respective regression lines for both size fractions are included in the panel. Data from the highly decayed cyanobacterial bloom at Station TF271 (gray symbols) were excluded from the regression analysis.

THAAN wt% TN concentrations in mesozooplankton ranged from 54 to 77 wt% TN and from 65 to 81 wt% TN in the small and large mesozooplankton size fractions, respectively, and were uncorrelated with the corresponding TN and TC concentrations or atomic C/N ratios (Supporting Data SD2_1). The highest THAAN wt% TN concentrations were mainly found in animals from the central Baltic Sea (73.2 ± 4.1 THAAN wt% TN), and the lowest values were mainly found at the coast near stations (66.1 ± 6.0 THAAN wt% TN, Supporting Data SD2_1). Interesting outliers were the low concentrations in both mesozooplankton size fractions in the highly decayed *Nodularia* bloom at Sta. TF271 in the eastern Gotland Basin (65.2 ± 4.9 THAAN wt% TN). Excluding the outliers at Sta. TF271, significant positive correlations between the cell carbon concentrations of Nostocales from the upper 10 m and the THAAN concentrations from both size fractions were found. These correlations further improved by averaging the Nostocales cell carbon data from the upper 20 m depth where available (Figure 2 and Supporting Data SD2_3).

### 3.2 Microplankton communities across the Baltic Sea

The cluster analysis based on the microplankton cell carbon concentrations in the upper 10 m (Supporting Data SD1_1) identified four different microplankton clusters across the Baltic Sea in summer (Supporting Figure S2), with the dominant taxa in each cluster identified by SIMPER analysis (Supporting Data SD1_2). Only the cluster in the central Baltic Sea (Stas. TF109, TF113, TF213, TF259, TF271) was dominated by N_2_‐fixing, filamentous cyanobacteria (namely, *Nodularia* and/or *Aphanizomenon*). The other three more near‐coastal clusters in the western Baltic Sea (Stas. TF360 ad TF12), in the upwelling area off Öland (Stas. UP2‐UP4), and in the upwelling area off Finland (Stas. UP5 and UP6) were codominated by different combinations of non‐N_2_‐fixing species, such as dinoflagellates, unicellular cyanobacteria, *Pseudanabaena*, small diatoms, or *Mesodinium rubrum* (Supporting Data SD1_2). The Shannon diversity (H’) and the Margalef's species richness (d) indices (Table 1) were significantly lower at the upwelling stations (average H’ of 3.0 ± 0.3; average d of 6.3 ± 0.5) than at the western and central Baltic Sea communities (average H’ of 3.7 ± 0.2; average d of 8.7 ± 0.9; two‐tailed Student's t tests: *p* = 2.6E^−3^, df = 6 and *p* = 2.7E^−4^, respectively).

### 3.3 Nitrogen source and trophic position proxies in mesozooplankton

**Figure 3:**
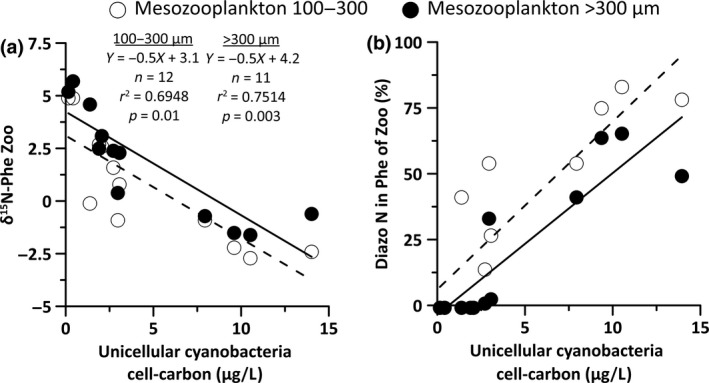
(a) The δ^15^N‐Phenylalanine (Phe) from two mesozooplankton size fractions relative to the cell carbon concentrations of unicellular cyanobacteria (μg L^−1^) averaged from the upper 10 m (UP stations) or upper 20 m (other stations). (b) Estimated share of Phe from diazotroph N in zooplankton based on a simple isotope mixing model.

The lowest δ^15^N‐Phe values in both mesozooplankton size fractions were found at the central Baltic Sea Stas. TF213, TF259, TF271, and TF284 (range of −2.6‰ to −0.5‰), while the highest δ^15^N‐Phe values were found in the western Baltic Sea at Stas. TF360 and TF12 (range of 5.0‰ to 5.8‰, Table 2). Interestingly, decreasing δ^15^N‐Phe values in mesozooplankton were significantly correlated with increasing cell carbon biomasses of non‐N_2_‐fixing, unicellular cyanobacteria. These correlations were further improved by including the unicellular cyanobacterial cell carbon data from the upper 20 m depth where available (Figure 3A, Supporting Data SD2_4). According to equation (1), essential Phe that received its nitrogen from N_2_ fixation (diazotroph Phe) contributed up to 84% and up to 65% of the Phe pool in the small and large mesozooplankton size fraction samples, respectively, in the central Baltic Sea (Figure 3B).

**Figure 4:**
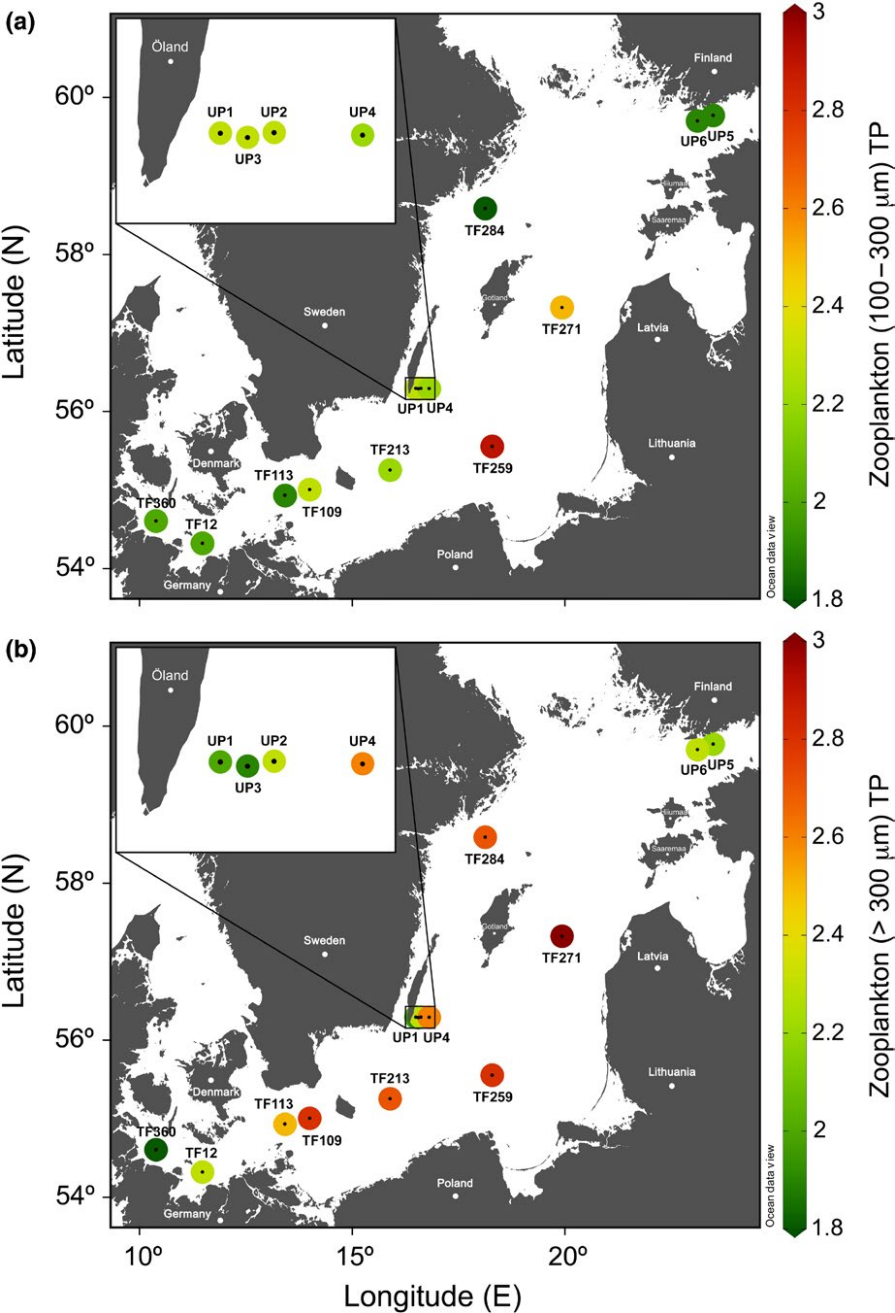
Distribution of the mean trophic positions (TPs) of the mesozooplankton size fractions 100–300 μm and >300 μm across the Baltic Sea in summer. Station names are shown below color‐coded TP estimates. TPs of 2.0 ± 0.3: herbivores; 3.0 ± 0.3: carnivores.

The TPs in the zooplankton communities ranged from 1.8 to 2.9 and covered the range from predominantly herbivorous to predominantly carnivorous communities across the Baltic Sea with distinct regional patterns (Figure 4). Zooplankton from the near‐coastal microplankton communities of the western Baltic, the upwelling area off Öland, and the upwelling area in the Gulf of Finland were predominantly herbivorous in both size fractions (Figure 4). In contrast, animals from the central Baltic Sea microplankton community became increasingly carnivorous from the Arkona Basin (Stas. TF113 and TF109) toward the Bornholm (TF213) and southern (Sta. TF259) and eastern (Sta. TF271) Gotland Basin stations (Figure 4). Animals in the larger size fraction were dominated by carnivores more often (TP >2.5) compared to the smaller size fraction, in which animals more often were predominantly herbivores (TP <2.5).

### 3.4 Statistical analyses to identify the environmental controls on the food web structure

**Figure 5:**
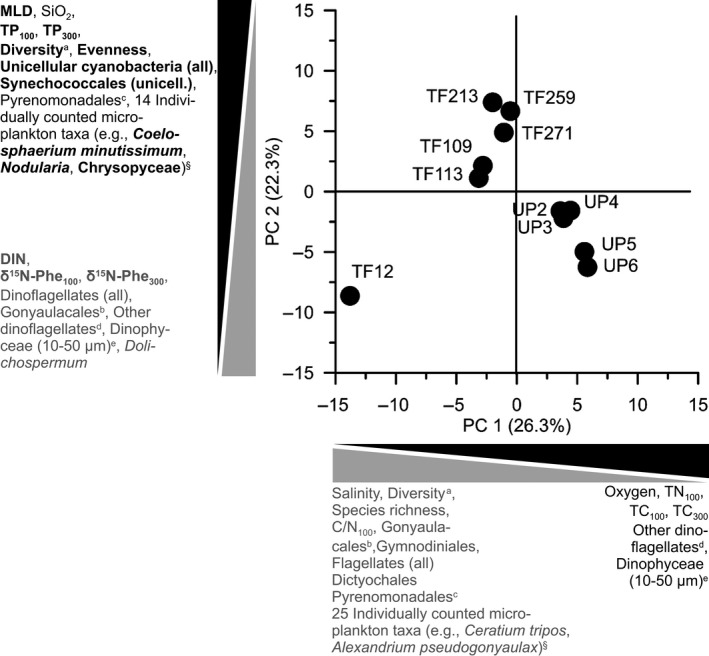
Factors regulating the planktonic food web structure and its nitrogen supply during blooms of unpalatable, filamentous, N_2_‐fixing cyanobacteria according to a two‐dimensional principal component analysis ordination of the first two leading principal components (PC 1: *x*‐axis; PC 2: *y*‐axis) of 124 environmental variables from the Baltic Sea in summer of 2015. All variables that dominated the eigenvectors of PC 1 and PC 2 are given along each axis. Variables directly involved in elucidating the environmental controls on the planktonic food web structure and the supply with nitrogen from N_2_ fixation are highlighted in bold. See text for more details. For biotic variables that were leading in both PCs, lowercase letters identify the leading abiotic variable(s) with most significant post hoc linear correlation(s): (a,c) mixed layer depth (MLD) and dissolved inorganic nitrogen (DIN), (b) salinity, or (d,e) DIN. ^§^see text and Supporting Data SD2_5 for all individually counted microplankton taxa.

A PCA was used to identify the environmental controls on the planktonic food web structure in the Baltic Sea during blooms of unpalatable, N_2_‐fixing, filamentous cyanobacteria. A total of four PCs were necessary to cover all abiotic variables from Table 1 that were included in the PCA (Supplemental Data SD2_5). Together, the four leading PCs explained 72.5% of the variability of the 124 variables from 11 stations that were included in the PCA (Supporting Data SD2_2). The eigenvalues of all PCs and the cumulative variance, as well as the loading components of the different variables making up all four PCs are shown in the Supporting Data SD2_5. Accordingly, PC 1 (26.3% of variability) was driven by decreasing salinity and to a lesser degree increasing oxygen concentrations, PC 2 (22.3% of variability) was driven by an increasing mixed layer depth and to a lesser degree increasing SiO_2_ concentrations at decreasing DIN concentrations, and PC 4 (PC 4: 10.6%) was driven by decreasing PO_4_^3−^ concentrations and to a lesser degree increasing temperature. Different from the other four PCs, PC 3 (13.3% of variability) was driven by the biotic variables for the (autocorrelated) biomasses of cyanobacteria (all) and filamentous cyanobacteria (Supporting Data SD2_5). While there is substantial variation explained by PC 3 and PC 4 (together explaining 23.9% of the variability), here we focused on PC 1 and PC 2 (together explaining 48.5% of the variability) to identify environmental controls specifically on the planktonic food web structure and its nitrogen supply across the Baltic Sea (Figure 5). The reason for this is that already PC 2 comprised both the mean trophic position and inorganic nitrogen source proxies for the two mesozooplankton size fractions as leading biotic variables, while the leading abiotic and biotic variables of PC 3 and PC 4 showed no significant relationship with the two proxies in either mesozooplankton size fraction (Supporting Data SD2_5).

Besides decreasing salinity and increasing oxygen concentrations, PC 1 generally represented an axis of decreasing microplankton diversity and species richness, as well as of mainly decreasing biomasses for a number of summarized microplankton groups of dinoflagellates and flagellates and multiple individually counted taxa (Figure 5, Supporting Data SD2_5). Among the 81 individually counted microplankton taxa, 25 mainly non‐N_2_‐fixing species had eigenvalues <−0.100 and showed positive correlations between their biomasses with salinity (*p* < 0.05) primarily due to their high biomasses at the high saline Sta. TF12 in the western Baltic Sea (see Supporting Data SD2_5 for all 28 individual species). Among the leading variables for mesozooplankton, TN and TC of the small mesozooplankton size fraction robustly increased with decreasing salinity (e.g., correlations not driven by data from a single station), while C/N ratios decreased. Interestingly, larger animals had constant C/N ratios, although their TC also increased with decreasing salinity (Supporting Data SD2_2 and SD2_5). In contrast to salinity, oxygen only had very weak relationships with the biotic variables in general (e.g., all correlations driven by data from a single station, Supporting Data SD2_5).

Besides increasing mixed layer depth and SiO_2_ concentrations, PC 2 generally represented an axis of increasing microplankton diversity and species evenness, as well as of increasing biomasses for a number of summarized microplankton groups of unicellular cyanobacteria and flagellates (Figure 5, Supporting Data SD2_5). Furthermore, the mean TPs of both mesozooplankton size fractions (TP_100_ and TP_300_) increased along PC 2. In contrast, DIN concentrations as well as biomasses for a number of summarized microplankton groups of dinoflagellates together with the δ^15^N‐Phe values as N source proxies from both mesozooplankton size fractions (δ^15^N‐Phe_100_ and δ^15^N‐Phe_300_) decreased along PC 2 (Supporting Data SD2_5, Figure 5). Notably, none of the dinoflagellate taxa with significant positive or negative relationships with mixed layer depth or DIN showed any significant relationship with the TP or N source proxies in mesozooplankton (Figure 5, Supporting Data SD2_5). In contrast to the mixed layer depth and DIN, SiO_2_ only had weak relationships with very few biotic variables (e.g., all correlations driven by data from a single station, Supporting Data SD2_5). Among the 81 individually counted microplankton taxa, 12 out of 16 taxa with high loadings for PC 2 (>ǀ100ǀ) showed significant (*p* < 0.05) and robust (e.g., correlations not driven by data from a single station) increases in their biomasses with increasing mixed layer depth and fewer also with decreasing DIN concentrations (5 taxa). Only 10 of these 12 taxa correlated significantly and mainly robustly with one or more of the two biogeochemical proxies for the two mesozooplankton size fractions, the majority of which belonged to the group of unicellular cyanobacteria (6 taxa, Supporting Data SD2_5). The most prominent taxon (e.g., >10% contribution to the average biomass in a microplankton community, Supporting Data SD1_2) among the other four taxa was *Nodularia*, which showed strong positive and negative relationships with the TP and δ^15^N‐Phe proxies, respectively, of the large mesozooplankton size fraction (Figure 5, Supporting Data SD2_5). Finally, its noteworthy that despite its comparatively low density (e.g., max. 1.55 μg cell carbon at Sta. TF 259), the individually counted, mixotrophic flagellate group of Chrysophyceae also showed consistent and mainly robust positive and negative relationships with the TP and δ^15^N‐Phe proxies, respectively, of both mesozooplankton size fractions at increasing mixed layer depth (Figure 5, Supporting Data SD2_5). In summary, among the different microplankton taxa, the N source and TP proxies of both mesozooplankton size fractions consistently decreased and increased, respectively, namely with at times very dense biomasses of unicellular cyanobacteria (e.g., total unicellular biomass of max. 17.98 μg cell carbon at Sta. TF 213) at increasing mixed layer depth.

## 4 DISCUSSION

The mean trophic position of zooplankton is routinely used in biogeochemical models (Butenschön et al., 2016; Daewel & Schrum, 2013; Hjøllo, Huse, Skogen, & Melle, 2012; Neumann, Fennel, & Kremp, 2002), and yet, the degree of complexity of planktonic food webs under different environmental conditions that can lead to changes in phytoplankton biomass is still not well understood (Maar et al., 2018; Peck et al., 2018). Our examination of the mean trophic position of field mesozooplankton using amino acid δ^15^N revealed how the structure of planktonic food webs can alter with changing environmental conditions. Coastal and offshore habitats in the Baltic Sea in summer provided a large variety of microplankton communities and planktonic food web structures. Our results in combination with complementary studies from the same cruise (Eglite et al., 2018; Loick‐Wilde et al., 2018) indicate that changes from herbivory to carnivory are underlain by changes in stratification, N_2_ fixation, microplankton diversity and composition, and cyanobacterial bloom stage. Hence, amino acid nitrogen stable isotope‐based mean trophic position and nitrogen source estimates in mesozooplankton provide empirical data to study the environmental controls that can lead to changes in the structure of planktonic food webs.

### 4.1 Stratification, nitrogen sources, and microplankton biomass and diversity

Salinity determined the distribution of many non‐N_2_‐fixing microplankton species with the most profound impact on dinoflagellate biomass. However, stratification regulated much of the availability of new nitrogen sources, the biomass of phytoplankton key species such as N_2_‐fixing *Nodularia* and non‐N_2_‐fixing unicellular cyanobacteria, as well as of microplankton diversity with ultimate consequences for the planktonic food web structure.

More eutrophic, near‐coastal sites with shallow mixed layer depths, for example, in the upwelling areas off Öland and in the Gulf of Finland, were in contrast to more DIN‐depleted areas in the central Baltic Sea, including the Arkona, Bornholm, and southern and eastern Gotland Basins with deeper mixed layer depths. In addition to the slightly lower DIN concentrations in the central Baltic Sea compared to the more coastal near sites, three additional lines of evidence suggested spatially different new nitrogen sources. First, direct evidence was given by the very high N_2_ fixation rates in the central Baltic Sea including the highly degraded *Nodularia* bloom in the eastern Gotland Basin (Loick‐Wilde et al., 2018), in contrast to the comparatively low N_2_ fixation rates in the upwelling area off Öland. Second, the microplankton community structure was dominated by N_2_‐fixing, filamentous cyanobacteria in the central Baltic Sea only, while at the near‐coastal sites, non‐N_2_‐fixing microplankton species dominated. Finally, the high values in the δ^15^N‐Phe of zooplankton as a time‐integrating proxy for the dominant N source in a planktonic food web confirmed preferential growth on nitrate at the near‐coastal sites. In contrast, the negative or low δ^15^N‐Phe values in the central Baltic Sea confirmed a preferential growth of the food webs on N_2_ fixation.

Despite the well‐described positive effect of salinity on the diversity of plankton species in general (Herlemann et al., 2011 and references therein), the depth of the mixed layer had a more profound impact on microplankton diversity than salinity in the Baltic Sea in summer (Supporting data SD2_2 and SD2_5). Most likely, this result was related to the blooms of positively buoyant, N_2_‐fixing, filamentous cyanobacteria that can substantially increase the diversity in microbial food webs (Hewson et al., 2009; Loick‐Wilde et al., 2017; Sheridan, Steinberg, & Kling, 2002). Accordingly, higher microplankton diversities were found at the central Baltic Sea stations compared to that in most of the near‐coastal stations that largely lacked N_2_‐fixing, filamentous cyanobacteria, even when salinity was higher at the near‐coastal stations (Table 1). Microplankton biomass and diversity were especially low at the upwelling stations despite enhanced nutrients, which is typical for recent upwelling events in the Baltic Sea (Nausch et al., 2009; Vahtera, Laanemets, Pavelson, Huttunen, & Kononen, 2005) and elsewhere (Blasco, Estrada, & Jones, 2013; MacIsaac, Dugdale, Barber, Blasco, & Packard, 1985).

Interestingly, microplankton diversity further increased from the Arkona toward the Bornholm and southern and eastern Gotland Basins (Table 1). Concomitantly, an increase in the age of the cyanobacterial blooms from the Arkona toward the Bornholm and southern and eastern Gotland Basins has been documented based on organic matter degradation proxies, phosphate concentrations, and visual inspections of the cyanobacterial cells in a complementary study (Loick‐Wilde et al., 2018). The underlying physical mechanism for both patterns was likely a transition from late‐summer stratification toward a wind‐ and thermal‐induced deepening of the mixed layer with the onset of the fall mixing that typically starts at the end of July and beginning of August in the central Baltic Sea (Leppäranta & Myrberg, 2009). This deepening of the mixed layer probably proceeded more in the Bornholm and southern and eastern Gotland Basins than in the Arkona Basin and must have contributed to an earlier breakdown of the *Nodularia* bloom in the eastern Gotland Basin. The critical role of stratification and water temperatures <16°C for the breakdown of cyanobacterial blooms, namely of *Nodularia*, in the Baltic Sea has earlier been observed (Kanoshina, Lips, & Leppänen, 2003; Wasmund, 1997). However, in contrast to many diatoms, senescent unpalatable filamentous cyanobacteria are positively buoyant as long as their gas vacuole is intact (Sellner, 1997), which results in the degradation of their biomass components, such as AAs in surface waters, namely, in the highly decayed *Nodularia* bloom in the eastern Gotland Basin (Loick‐Wilde et al., 2018). The advancing decay of cyanobacterial cells, including a growing availability of their amino acids for the surrounding heterotrophs in surface waters, must have additionally fostered the complexity of the microbial food webs from the Arkona toward the Bornholm, and southern and eastern Gotland Basins with large consequences for the mean trophic positions of mesozooplankton.

### 4.2 Planktonic food web structure across the Baltic Sea in summer

Mesozooplankton across the Baltic Sea covered a large range of mean trophic positions from dominantly herbivorous animals at the near‐coastal sites to dominantly carnivorous animals in the central Baltic Sea, which points to fundamentally different factors regulating the food web structure during blooms of unpalatable, N_2_‐fixing, filamentous cyanobacteria compared to palatable, non‐N_2_‐fixing microplankton communities.

Herbivory in mesozooplankton at the near‐coastal sites probably indicates that mesozooplankton directly grazed on the nitrate‐grown primary producers. In contrast, filamentous, N_2_‐fixing cyanobacteria are hardly grazed directly (Loick‐Wilde et al., 2012; Mulholland, 2007; Wannicke et al., 2013). Their exuded ammonium or DON for growth is taken up by unicellular cyanobacteria that do not fix N_2_ in the Baltic Sea and from which the diazotroph nitrogen can be directly incorporated by mesozooplankton species (Motwani et al., 2018; Stal et al., 2003). Three lines of evidence support the idea that generally, this trophic relationship was also responsible for the incorporation of diazotroph nitrogen into mesozooplankton during our study. First, the group of Nostocales, namely, N_2_‐fixing, unpalatable, filamentous *Nodularia* and *Aphanizomenon,* were responsible for the high N_2_ fixation rates in the microplankton community in the central Baltic Sea according to significant positive correlations of their cell carbon biomasses with N_2_ fixation rates (Supporting Data SD2_1). Second, the negative δ^15^N‐Phe values in mesozooplankton in the nitrate‐depleted waters of the central Baltic Sea stations provide strong evidence for the intensive incorporation of essential Phe that must have been synthesized from diazotroph ammonium. Third, the δ^15^N‐Phe values in both mesozooplankton size fractions decreased with increasing cell carbon biomasses of unicellular cyanobacteria but not with *Nodularia* and *Aphanizomenon* cell carbon biomasses (Figure 2A). According to the simple isotope mixing model, diazotroph Phe accounted for a maximum of 83.9% and 66.1% of the essential Phe pool in the small and large mesozooplankton size fraction, respectively, when unicellular cyanobacteria were abundant. These numbers are at the high end of the contribution of diazotroph N to total nitrogen in mesozooplankton from the oligotrophic, tropical North Atlantic, where diazotroph N contributed a maximum of 65–67% to the organic nitrogen pool in mesozooplankton (Montoya et al., 2002). The high contribution of diazotroph Phe in animal tissues related to high unicellular cyanobacterial biomass further stresses the vector function of unicellular cyanobacteria for the supply of essential diazotroph amino acids for higher trophic levels in the Baltic Sea (Karlson et al., 2015; Loick‐Wilde et al., 2012; Motwani et al., 2018; Paul, Sommer, Garzke, Moustaka‐Gouni, Paul et al., 2016).

**Figure 6:**
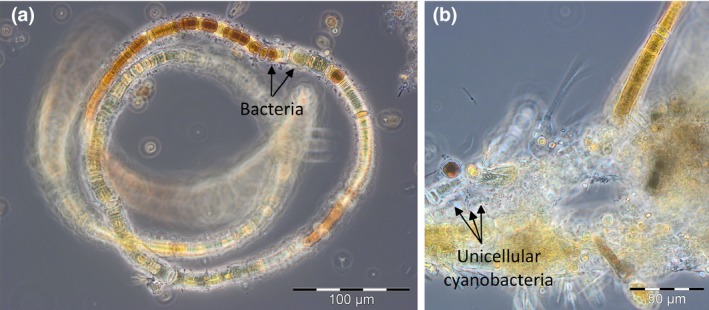
Biocenoses associated with the highly decayed *Nodularia* bloom in the mixed layer of the eastern Gotland Basin, including (a) unidentified bacteria and (b) unicellular cyanobacteria.

Interestingly, the mean trophic positions of mesozooplankton from the central Baltic Sea were not homogenously carnivorous; rather, spatial and zooplankton size‐specific differences occurred. In the Arkona Basin (Stas. TF113 and TF109) and Bornholm Basin (Sta. TF213), herbivorous TPs in animals from the smaller mesozooplankton‐sized fractions indicate that smaller animals must have intensively grazed directly on co‐occurring autotrophs such as unicellular cyanobacteria during the younger N_2_‐fixing cyanobacterial blooms. In contrast, TPs of 2.9 and 2.5 for small mesozooplankton in the southern (Sta. TF259) and eastern Gotland Basins (Sta. TF271), respectively, indicate that smaller animals must have predominantly preyed on mixo‐ and heterotrophic microorganisms in the more decayed cyanobacterial blooms (Figure 2). Additionally, larger animals showed a clear increase in their mean trophic position with aging cyanobacterial blooms according to a predominantly omnivorous feeding behavior in the Arkona Basin (Sta. TF113) and toward a predominantly carnivorous feeding behavior in the eastern Gotland Basin. In the southern and eastern Gotland Basins, carnivorous animals from both size fractions must have intensively preyed on diverse microbial biocenoses of bacteria, unicellular cyanobacteria, and mixo‐ and heterotrophic flagellates that had developed in association with the positively buoyant, aging or decaying *Nodularia* colonies (Figure 6).

A common theme from bulk carbon and nitrogen pools in mesozooplankton was that larger animals rather than smaller animals in general were better able to keep body stoichiometry constant across the salinity gradient. The underlying causes for the variation in the C:N ratios with salinity in the smaller animals are potentially manifold (Steinberg & Saba, 2008) and may include changes in the mesozooplankton community structure (Walve & Larsson, 1999), changes in the lipid pools (Gismervik, 1997), or changes in ammonium excretion and growth efficiency (Checkley & Entzeroth, 1985; Koski, 1999; Walve & Larsson, 1999). In contrast, the metabolic nitrogen pool in mesozooplankton did not change with salinity but primarily with the biomass of N_2_‐fixing Nostocales outside the predominantly decayed bloom in the eastern Gotland Basin. An increase in the metabolic nitrogen pools of mesozooplankton due to the intensive incorporation of diazotroph amino acids from *N. spumigena* at constant C:N ratios has previously been observed in field mesozooplankton from the central Baltic Sea (Loick‐Wilde et al., 2012). This supports the idea that the amino acid nitrogen pool is a sensitive measure to quantify the impact of unpalatable, filamentous, N_2_‐fixing cyanobacterial blooms on mesozooplankton physiology. In the decayed bloom, the size of the metabolic nitrogen pools in carnivorous mesozooplankton was more similar to the small pools in the near‐coastal herbivorous animals. Thus, we can only speculate that differences in the turnover times of AAs associated with food quality were responsible for the relatively small metabolic nitrogen pools in mesozooplankton, which deserves further investigation.

Our data support the prevailing view that direct grazing of mesozooplankton on palatable phytoplankton species that co‐occur with the unpalatable, filamentous, N_2_‐fixing species dominates in a highly stratified, nitrate‐depleted water column. However, in association with the increasing decay of blooms of unpalatable N_2_‐fixing cyanobacteria, carnivory became the dominant feeding mode in mesozooplankton, which stresses the at times important role of heterotrophic microbial food webs for the planktonic food web structure. In the next step, empirical data of the mean trophic position of mesozooplankton and the dominant new nitrogen source for biological production will be used to calibrate and validate current biogeochemical models, including end‐to‐end models (e.g., physics to fish to human sectors, Peck et al., 2018).

Finally, an increase in sea surface stratification linked to sea surface warming, which tends to slow the nutrient supply to the surface, is projected for future oceans (Gattuso et al., 2015; Roy et al., 2011). Under these conditions, unpalatable N_2_‐fixing *Trichodesmium* are especially favored, and their large blooms are expected to further expand under future global warming scenarios because enhanced N_2_ fixation was found to persist under high CO_2_ irrespective of phosphorus limitation (Hutchins & Fu, 2017; Hutchins et al., 2017; Walworth et al., 2018). Analogously to *Nodularia* blooms in the Baltic Sea (Wasmund, Nausch, & Voss, 2012), blooms of *Trichodesmium* in other marine systems develop seasonally in association with mixing of DIN‐depleted but phosphorus‐rich upwelling waters into warm, stratified oceanic waters that contain seed populations of *Trichodesmium* (Deutsch, Sarmiento, Sigman, Gruber, & Dunne, 2007; Hegde et al., 2008; Hood, Coles, & Capone, 2004). Further, tropical and subtropical *Trichodesmium* blooms also decay in surface waters, which is namely triggered by the deepening of the mixed layer depth (Devassy, Bhattathiri, & Qasim, 1979; Hood et al., 2004) as found for *Nodularia* blooms (Kanoshina et al., 2003; Wasmund, 1997). It is a testable hypothesis that the observed changes in the planktonic food web structure in the Baltic Sea also take place in decaying *Trichodesmium* blooms in other marine systems, which would imply that a carnivorous feeding behavior in mesozooplankton can become more common under future global warming scenarios.
