# Supplementary material for: Stratification, nitrogen fixation, and cyanobacterial bloom stage regulate the planktonic food web structure
Source: Glob Chang Biol. 2019 Jan 9;25(3):794–810. doi: 10.1111/gcb.14546 (PMC6850720; doi:10.1111/gcb.14546)
Supplement: Supplementary file 1 [file GCB-25-794-s001.doc]

**Supporting Material 1**

*Elemental and biochemical analyses of plankton samples*

Frozen particle samples on filters and zooplankton samples were dried for 48 h at 60 ºC, and weighted to determine dry weight (DW) biomass. GF/F filters were wrapped in tin capsules, whereas those samples containing zooplankton were first grounded to fine powder and then subsampled (∼1 mg DW) in tin capsules. Suspended POM on GF/F filters and zooplankton subsamples were subsequently analyzed for elemental concentrations (i.e., TN and TC) and bulk stable isotope abundances (i.e., bulk *δ*^13^C and *δ*^15^N) by means of Elemental Analysis-Isotope Ratio Mass Spectrometry (EA-IRMS). POM retained on the Nuclepore filters (usually between 5-10 mg of dry particulate material) and additional zooplankton subsamples (∼ 20 mg DW each) were assayed for AA composition and compound-specific amino acid isotope ratios (CSI-AA). To make individual AAs more volatile, less reactive, and thus improve their chromatographic behavior, samples were derivatized into TFA-isopropyl esters as described elsewhere (Hofmann et al. 2003, Eglite et al. 2018). The resulting TFA-derivatives were finally stored in dichloromethane at −20 ºC awaiting analysis both in the gas chromatography-mass spectrometer (GC-MS) and in the gas chromatography-combustion-isotope ratio mass spectrometer (GC-C-IRMS).

Individual concentrations of 13 AAs were measured in the GC-MS and included the so-called “source” AAs glycine -Gly-, lysine -Lys-, phenylalanine -Phe- and serine -Ser-, the “trophic” AAs alaline -Ala-, aspartic acid -Asp-, glutamic acid -Glu-, isoleucine -Ile-, leucine -Leu-, proline -Pro-, tyrosine -Tyr- and valine -Val-, and the “metabolic” AA threonine -Thr-, categorized as such, among others, by Germain et al. (2013), McClelland and Montoya (2002) and Chikaraishi et al. (2009) according to the sensitivity of each AA to trophic enrichment in ^15^N. It should be noted that Asp and Glu also include the amide forms asparagine and glutamine, respectively. Measurements were carried out in a DANI gas chromatograph/time-of-flight mass spectrometer (DANI Instruments SA, Italy).

All samples were further analyzed in triplicates for CSI-AA using a Thermo Scientific GC coupled to a Thermo MAT 253 IRMS via a Thermo GC IsoLink and ConFlo IV interface (Thermo Fisher Scientific, Germany). The standard deviation between replicates did not exceed 1.0 ‰.

**Supporting Figure S1.** Correlation between discrete Chl. a measurements (mg m^-3^) and water column sensor data (rel. units). The regression statistic for regression line is included in the panel.

**Supporting Figure S2.** Cluster analysis of the phytoplankton communities A) in the upper 10 m depth, B) in the upper 10 m and at 20m depth, and C) averaged from the upper 10 m and at 20m depth for across the Baltic Sea. In all data sets, four clusters were identified at a similarity level of 28% including the following stations: western Baltic including TF12, TF360; central Baltic including TF109, TF113, TF213, TF259, TF271; upwelling off Öland including UP2, UP3, UP4; upwelling Gulf of Finland including UP5 and UP6. See text for more details.

A)

B)

C)

**Supporting Figure S3.** **Upper panel:** The composite relationships between the trophic position estimates via δ^15^N of glutamic acid –Glu- and phenylalanine –Phe- (TP_Glu/Phe_) and via δ^15^N of Alanine –Ala- and Phe (TP_Ala/Phe_) in **A)** the small (100-300 µm) and **B)** large (>300 µm) mesozooplankton size fractions. **Lower panel:** The composite relationships between TP_Glu/Phe_ and the heterotrophic microbial resynthesis proxy (ΣV) in this data set (Cross Baltic Data) in comparison to mesozooplankton data sets with intensive microbial re-synthesis from the suboxic zone of the Baltic Sea (Eglite et al. 2018) and no microbial resynthesis from the tropical North Pacific (Mompéan et al. 2016) in **C)** the small and **D)** large mesozooplankton size fractions. Close to 1:1 relationships between TP_Glu/Phe_ vs TP_Ala/Phe_  (including an outlier marked in red in panel B at station UP1) indicate no significant differences between either TP estimation approach. ΣV values of maximum 2 and coupled ΣV and TP values indicate the absence of intensive heterotrophic microbial re-synthesis of amino acids (Ohkouc*hi et a*l., 2017).
